# Association between dietary consumption of fatty acids and age-related macular degeneration in the National Health and Nutrition Examination Survey

**DOI:** 10.1038/s41598-024-61833-6

**Published:** 2024-05-14

**Authors:** Bingcai Jiang, Xin Wei, Dongmei Cai, Xiaoqin Wang, Xiaobo Zhou, Feng Chen, Xiaopeng Shen, Xiaochuan Cao, Changwei Zheng

**Affiliations:** 1 Department of Ophthalmology, Guizhou Provincial People’s Hospital, Guizhou, China; 2 Department of Ophthalmology, The People’s Hospital of Tongliang District, Chongqing, China

**Keywords:** Age-related macular degeneration, Saturated fatty acid, Polyunsaturated fatty acid, Monounsaturated fatty acid, Dietary intake, Visual system, Eye diseases

## Abstract

The aim of this study is to assess the relationship between dietary intake of fatty acids and the age-related macular degeneration (AMD) in the United States population. Adult participants of the 2005–2008 National Health and Nutrition Examination Survey (NHANES) were included in this nationwide cross-sectional study. Dietary fatty acid intake was obtained from two 24-h dietary recall interviews. The intake of dietary fatty acids was analyzed as a continuous and categorical variable. AMD status was assessed using nonmydriatic fundus photographs. Univariate and multivariate logistic regression analyses were used to assess the association between dietary fatty acid intake and AMD. The unweighted population included 4702 individuals of whom 374 had AMD. After adjusting for relevant variables, each 1 unit increase (1 mg/1000 kcal) intake of EPA (OR: 0.996, 95% CI: 0.993–0.996, P = 0.018), DPA (OR: 0.976, 95% CI: 0.962–0.990, P = 0.002), and DHA (OR: 0.996, 95% CI: 0.994–0.999, P = 0.003) were significantly decreased odds of any AMD. The highest versus lowest quartile of EPA (OR: 0.476, *P* for trend < 0.001), DPA (OR: 0.467, *P* for trend = 0.005) and DHA (OR: 0.586, *P* for trend = 0.008) were negatively associated with the odds of any AMD. Subgroup analysis showed that higher quartiles of EPA (OR: 0.461, *P* for trend < 0.002), DPA (OR: 0.467, *P* for trend = 0.006) and DHA (OR: 0.578, *P* for trend = 0.007) exhibited a negative association with early AMD. The study found no significant association between the intake of dietary fatty acids, including n-3 PUFA, and the odds of late AMD. In the 2005–2008 NHANES population, higher dietary DHA, DPA and EPA intake associated with decreased odds of early AMD. However, no clear association was found between specific types of FAs and late AMD.

## Introduction

Age-related macular degeneration (AMD) is the third leading cause of visual impairment worldwide^1^. Among adults aged 45–85 years, the prevalence of AMD is 8.7%, and the number of global population will increase to 288 million by 2040^2^. Based on fundus photography findings, AMD can be divided into early and late AMD. Early AMD is characterized by moderate drusen or retinal pigment abnormalities. Late AMD includes two forms: geographic atrophy (GA) and neovascular AMD. Although intravitreal injection of anti-VEGF drugs may have benefits in restoring lost vision for neovascular AMD, they remain less effective in the long term^3^. However, no treatments are clinically available for GA. Hence, preventative approaches are attractive for AMD progression.

AMD is a complex disease that interplays between genetics and environmental factors, including dietary factors^4,5^. Fatty acids (FAs), as a major type of dietary component, have gained significant attention due to their numerous effects on human health^6^. The retina is one of the most lipid-rich tissues in the human body^7^. Among retinal phospholipids, polyunsaturated fatty acids (PUFAs) account for 45% of total phospholipids, saturated fatty acids (SFAs) account for 37%, and monounsaturated fatty acids (MUFAs) account for 10%^8^. In recent decades, there has been growing interest in the role of FAs, especially the n-3 PUFAs, in the pathogenesis and prevention of AMD^9–11^. Clinical and epidemiologic studies have shown that a higher intake of n-3 PUFAs is associated with a decreased risk of AMD^11–15^ and that n-6 PUFAs are associated with an increased AMD risk^16^. However, the landmark age-related eye disease Study 2 (AREDS2) reported that n-3 PUFAs supplementation does not benefit patients with AMD^17^. A previous randomized intervention trial (Nutritional AMD Treatment-2) also suggested patients with early lesions of AMD indicated no significant difference in drusen progression and neovascular after oral supplementation with n-3 PUFAs^18^. In addition, studies of the associations between individual MUFAs or SFAs and AMD risk are few, and the results have been inconsistent^19–21^.

While most epidemiological findings support a beneficial effect of n-3 PUFAs on AMD, randomized controlled trials (RCTs) did not observe a reduced risk of advanced AMD. Additionally, existing studies give less consideration to the wider range of individual FA intake that are part of the human diet. So, there is a need for more epidemiological studies of wider range of FAs and their relationship to any AMD. Hence, we undertook this nationwide cross-sectional study from the National Health and Nutrition Examination Survey (NHANES) 2005–2008 to evaluate the association between individual FA intake and any AMD in adults residing in the United States. In the study, the intake of 19 dietary FAs, including the total of SFAs, MUFAs and PUFAs, was calculated as energy density (kcal) separately (mg/1000 kcal).

## Methods

### Study design and population

**Figure 1:**
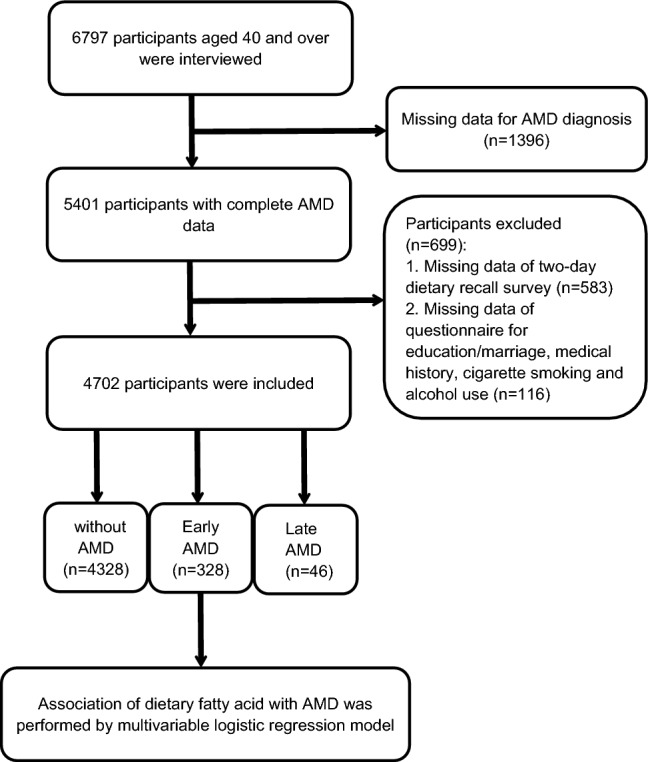
Flowchart of the study.

The NHANES is a comprehensive survey that assesses the health and nutritional status of noninstitutionalized individuals in the United States. It utilizes a stratified multistage probability sample design to ensure national representation. NHANES conducts home interviews and medical examinations at mobile medical centers (MECs) and further collects information on sociodemographics, lifestyle, dietary intake, behavior and medical conditions. The data are released every two years. This cross-sectional study encompassed two waves of NHANES, namely 2005–2006 and 2007–2008. In the NHANES during the two circles, individuals who had eye patches, eye infection or blindness were excluded. A total of 6797 individuals aged 40 years or older took retinal photographs. Of these, 1396 individuals were excluded on account of the absence of AMD diagnostic data. A total of 699 participants who had incomplete data were excluded, as shown in Fig. 1. Ultimately, 4702 (2305 males and 2397 females) participants were included in the study. It is important to note that NHANES data are publicly available for researchers, and the NHANES administration is approved by the National Center for Health Statistics (NCHS) Ethics Review Board. The research was performed in accordance with the tenets of the Declaration of Helsinki. All data used in this study are available in NHANES website: https://www.cdc.gov/nchs/nhanes/ and in supplementary file.

### Diagnosis of AMD

Retinal images were captured using a Canon CR6-45NM Ophthalmic Digital Imaging System and a digital camera (EOS 10D; Canon USA, Inc.) during the NHANES survey from 2005 to 2008. These AMD categories are available under the “OPDDARM” and “OPDSARM” variables in the NHANES. All fundus images were graded at the University of Wisconsin-Madison, according to the modified Wisconsin Age-Related Maculopathy Grading Classification Scheme^22^. Early AMD was classified as the presence of drusen with a grid area larger than a 500-μm circle and/or pigmentary abnormalities, while signs of exudative or GA were considered as late AMD. All images were graded by at least 2 trained examiners, while a third senior examiner could adjudicate any disagreements. If both eyes had retinal images, the eye with the more severe status was adopted in the analyses.

### Dietary fatty acid intake

The main focus in this study was the daily intake of dietary FA, which was assessed using two 24-h dietary recall interviews conducted in the NHANES survey. The initial dietary recall interview took place at MECs, while the subsequent one was conducted via phone call 3 to 10 days later. The US Department of Agriculture (USDA) Food and Nutrient Database for Dietary Studies was used for processing the dietary intake values. An estimate of the daily aggregates of daily food nutrients and components (including SFAs, MUFAs and PUFAs) were recorded on each interview day, according to daily food consumption. The mean value of the two interview days was used in these analyses.

### Covariates

Based on the literature, a range of variables regarding demographic factors, lifestyles, and medical conditions that could be associated with the outcomes were selected as potential confounders^23,24^. Demographic factors included age, gender, ethnicity, education level, and marital status. Ethnicity was categorized as Mexican American, non-Hispanic black, non-Hispanic white, or other. The level of education was divided into two categories according to whether they had attended high school. The marital status was examined as a binary categorical variable with two levels: married/with a partner and unmarried/other. Lifestyle factors included smoking and alcohol consumption. Smoking status and alcohol consumption were categorized as yes or never. Those who smoked more than 100 cigarettes in their lifetime were defined as smokers and the rest were defined as never smoker. Those who consumed fewer than 12 drinks in lifetime were defined as never drink and the rest were defined as drinkers. Medical conditions included BMI, hyperlipidemia, diabetes mellitus (DM), hypertension, and cardiovascular disease (CVD). Hyperlipidemia and cardiovascular disease defined by subject’s self-report. BMI was calculated as weight divided by the square of height in meters. DM was defined as 2-h plasma glucose ≥ 200 mg/dL, hemoglobin A1C ≥ 6.5%, fasting plasma glucose ≥ 126 mg/dL^25^, taking insulin or diabetic medicine. Hypertension was defined as mean systolic blood pressure ≥ 130 mmHg or mean diastolic blood pressure ≥ 80 mmHg^26^, or taking antihypertensive medicine.

### Statistical analysis

Baseline characteristics of the study population were analyzed with descriptive statistics. The means (standard error of mean, SE) are used to present continuous data, while numbers (weighted percentages) are used to present categorical data. Participants were diagnosed with no, early or late AMD. For comparison of variables between groups, the weighted Chi-square test was used for categorical variables, and the weighted *t*-test was used for continuous variables. The weighted *t*-test is implemented by the svyttest command. The weighted Chi-square test is implemented by the svychisq command.

We conducted univariate and multivariate logistic regression to analyze the association between FAs and AMD. FA intake was analyzed as a continuous variable per 1 unit increase, and as a categorical variable divided into quartiles that reduce the influence of outliers on the regression results. When FA intake was divided into four intervals, the lowest quartile served as the reference group. Tests for linear trends were analyzed using logistic regression with median intake in each quartile of FAs as continuous variables. In the multivariate logistic regression analysis, the model was fully adjusted for age, sex, ethnicity, marital status, educational level, BMI (continuous), DM, hypertension, hyperlipidemia, CVD, smoking, alcohol, and total energy intake. Finally, we used restricted cubic spline to evaluate the potential non-linear relationships between dietary FA consumption and odds of early AMD.

The evaluation indicators were odds ratios (ORs) and 95% confidence intervals (CIs). All statistical tests were two-sided. A two-sided *P* value < 0.05 was considered as statistically significant. Statistical analyses were performed using R software (version 4.1.1) with the R package “survey”. Given the stratified multistage probability sample design, two-day dietary sampling weights were adopted in all analyses to ensure nationally representative estimates.

## Results

### Baseline characteristics

The Table 1 displays the baseline characteristics of the study population according to any AMD. A total of 111,845,463 weighted and 4702 unweighted participants, including 2305 (weighted 45.33%) males and 2397 (weighted 54.64%) females, were enrolled in the study. A total of 374 unweighted participants were diagnosed with AMD. Among the 374 AMD participants, 328 persons were diagnosed with early AMD, and the other 46 participants were diagnosed with late AMD (Fig. 1). The overall prevalence of any AMD was 7.95% with a mean age of 68.51 (0.92) years. The most common ethnicity was non-Hispanic White, including 2632 (weighted 78.57%) participants. There was no significant difference in BMI between non AMD group (29.12 ± 0.19) and AMD group (28.75 ± 0.53). There were significant differences between participants with and without AMD in age, energy intake, ethnicity, marital status, smoking, alcohol, hyperlipidemia, diabetes, hypertension and CVD.

**Table 1:** Characteristics of the study population (N = 4702, representing a weighted sample of 111,845,463).

Variables	Total (n = 4702)	Without AMD (n = 4328)	AMD (n = 374)	*P* value
Age, mean ± SE	56.55 (0.40)	55.69 (0.38)	68.51 (0.92)	< 0.0001
Gender, n (weighted %)				0.55
Female	2397 (54.64)	2207 (54.53)	190 (56.63)	
Male	2305 (45.33)	2121 (45.47)	184 (43.37)	
Energy intake, kcal, mean ± SE	2030.91 (21.08)	2045.50 (20.60)	1829.29 (61.36)	< 0.001
BMI, kg/m^2^, mean ± SE	29.10 (0.19)	29.12 (0.19)	28.75 (0.53)	0.49
BMI subgroups, n (weighted %)				0.6
< 25 kg/m^2^	1178 (27.10)	1077 (27.03)	101 (27.98)	
25 to < 30 kg/m^2^	1716 (36.35)	1567 (36.19)	149 (38.59)	
> = 30 kg/m^2^	1808 (36.55)	1684 (36.78)	124 (33.44)	
Ethnicity, n (weighted %)				< 0.001
Mexican American	698 (5.19)	656 (5.27)	42 (4.09)	
Non-Hispanic Black	918 (9.38)	887 (9.80)	31 (3.61)	
Non-Hispanic White	2632 (78.57)	2356 (77.85)	276 (88.48)	
Other	454 (6.86)	429 (7.08)	25 (3.82)	
Marital status, n (weighted %)				0.001
Married/with a partner	3055 (68.86)	2850 (69.64)	205 (58.03)	
Unmarried and other	1647 (31.14)	1478 (30.36)	169 (41.97)	
Education level, n (weighted %)				0.17
High school and over	4105 (94.06)	3788 (94.30)	317 (90.78)	
Less than high school	597 (5.94)	540 (5.70)	57 (9.22)	
Smoke, n (weighted %)				0.01
Yes	2472 (51.53)	2260 (50.85)	212 (61.03)	
Never	2230 (48.47)	2068 (49.15)	162 (38.97)	
Alcohol, n (weighted %)				0.02
Yes	4060 (89.33)	3757 (89.66)	303 (84.77)	
Never	642 (10.67)	571 (10.34)	71 (15.23)	
Hyperlipidemia, n (weighted %)				0.02
Yes	3736 (80.57)	3426 (80.16)	310 (86.35)	
No	966 (19.13)	902 (19.84)	64 (13.65)	
DM, n (weighted %)				0.002
Yes	1553 (26.49)	1402 (25.66)	151 (38.04)	
No	3149 (73.51)	2926 (74.34)	223 (61.96)	
Hypertension, n (weighted %)				< 0.0001
Yes	2553 (49.42)	2289 (48.19)	264 (66.36)	
No	2149 (50.58)	2039 (51.81)	110 (33.64)	
CVD, n (weighted %)				< 0.0001
Yes	707 (11.53)	594 (10.42)	113 (26.85)	
No	3995 (88.47)	3734 (89.58)	261 (73.15)	

*AMD* age-related macular degeneration, *DM* diabetes mellitus, *CVD* cardiovascular disease.

### Association between dietary fat acids and AMD

The results of the univariate logistic regression analyses assessing the association between specific types of dietary FA intake and any AMD are shown in Table 2. In terms of energy density (mg/1000 kcal), the mean Intakes of PUFAs 20:5 (EPA), PUFAs 22:5 (DPA) and PUFAs 22:6 (DHA) were higher in the non AMD group than in the any AMD group. The average energy intake of non AMD group and AMD group is 2045.50 and 1829.29 kcal, respectively. Based on average energy intake, the mean daily intake of EPA, DPA and DHA in non AMD group were 54.57, 20.86 and 99.3 mg/d. While the mean daily intake of EPA, DPA and DHA in AMD group were 29.15, 10.98 and 54.04 mg/d. WHO supports recommended intake levels of EPA + DHA of 400–1000 mg/d^27^. In this study, the intake of EPA + DHA in the non-AMD group was 153.87 mg/d, and that in the AMD group was 83.19 mg/d, which was lower than the recommended intake levels by WHO.

**Table 2:** Univariate logistic regression analysis of factors associated with the odds of any AMD.

Variables	Total	Without AMD	AMD	OR (95% CI)	P value
Energy density (mg/1000 kcal, mean ± SE)					
Total SFA	12,619.015 (77.180)	12,607.069 (76.520)	12,784.121 (320.699)	1.000 (1.000, 1.000)	0.582
SFA 4:0 (Butanoic)	274.473 (4.918)	273.621 (5.162)	286.248 (15.918)	1.000 (0.999, 1.001)	0.445
SFA 6:0 (Hexanoic)	146.790 (2.494)	146.093 (2.584)	156.425 (9.315)	1.001 (0.999, 1.003)	0.269
SFA 8:0 (Octanoic)	121.424 (1.965)	121.668 (2.067)	118.047 (6.089)	1.000 (0.998, 1.001)	0.591
SFA 10:0 (Decanoic)	219.055 (3.237)	218.310 (3.394)	229.352 (12.737)	1.001 (0.999, 1.002)	0.399
SFA 12:0 (Dodecanoic)	391.596 (9.363)	393.172 (9.773)	369.812 (28.419)	1.000 (1.000, 1.000)	0.476
SFA 14:0 (Tetradecanoic)	1057.358 (13.661)	1055.624 (13.798)	1081.324 (48.511)	1.000 (1.000, 1.000)	0.597
SFA 16:0 (Hexadecanoic)	6770.698 (36.610)	6764.810 (36.734)	6852.083 (144.097)	1.000 (1.000, 1.000)	0.550
SFA 18:0 (Octadecanoic)	3247.745 (22.269)	3244.819 (22.041)	3288.183 (82.134)	1.000 (1.000, 1.000)	0.595
Total MUFA	13,978.742 (74.100)	13,979.301 (74.847)	13,971.017 (252.194)	1.000 (1.000, 1.000)	0.974
MUFA 16:1 (Hexadecenoic)	585.349 (5.090)	586.358 (5.277)	571.408 (16.785)	1.000 (0.999, 1.000)	0.408
MUFA 18:1 (Octadecenoic)	13,050.741 (69.049)	13,049.413 (70.203)	13,069.086 (239.483)	1.000 (1.000, 1.000)	0.936
MUFA 20:1 (Eicosenoic)	116.864 (1.548)	116.766 (1.529)	118.210 (5.171)	1.000 (0.998, 1.002)	0.765
MUFA 22:1 (Docosenoic)	20.076 (0.973)	20.024 (0.913)	20.790 (5.485)	1.000 (0.998, 1.003)	0.877
Total PUFA	8309.835 (62.381)	8322.911 (60.830)	8129.123 (216.265)	1.000 (1.000, 1.000)	0.377
PUFA 18:2 (Octadecadienoic,n-6, LA)	7321.322 (57.519)	7329.709 (55.608)	7205.415 (200.448)	1.000 (1.000, 1.000)	0.532
PUFA 18:3 (Octadecatrienoic, n-3, ALA)	737.942 (8.186)	738.246 (8.321)	733.749 (21.959)	1.000 (1.000, 1.000)	0.841
PUFA 18:4 (Octadecatetraenoic,n-3, SDA)	7.337 (0.434)	7.471 (0.469)	5.474 (0.680)	0.990 (0.981, 1.000)	0.051
PUFA 20:4 (Eicosatetraenoic, n-6, AA)	68.662 (1.160)	69.223 (1.232)	60.917 (2.078)	0.995 (0.992, 0.998)	0.003
PUFA 20:5 (Eicosapentaenoic, n-3, EPA)	25.897 (1.812)	26.618 (1.917)	15.932 (2.121)	0.996 (0.993, 0.999)	0.006
PUFA 22:5 (Docosapentaenoic, n-3, DPA)	9.894 (0.464)	10.176 (0.490)	5.996 (0.491)	0.971 (0.958, 0.985)	< 0.001
PUFA 22:6 (Docosahexaenoic, n-3, DHA)	47.166 (2.713)	48.443 (2.887)	29.527 (2.662)	0.996 (0.994, 0.998)	< 0.001

When fatty acid intake was analyzed as a continuous variable, examination of the relationship between fatty acid subclasses and any AMD showed a negative association between PUFAs 20:4 (OR: 0.995, *P* = 0.003), EPA (OR: 0.996, *P* = 0.006), DPA (OR: 0.971, *P* < 0.001), and DHA (OR: 0.996, *P* < 0.001) and the odds of any AMD (Table 2), while the other 15 specific types of FAs were not related to the odds of any AMD. After fully adjusting for age, sex, ethnicity, BMI (continuous), smoking, alcohol, energy intake, marital status, education and medical history, EPA (OR: 0.996, 95% CI: 0.993–0.996, *P* = 0.018), DPA (OR: 0.976, 95% CI: 0.962–0.990, *P* = 0.002), and DHA (OR: 0.996, 95% CI: 0.994–0.999, *P* = 0.003) were still significantly associated with the odds of any AMD (Table 3). When analyzed in quartiles, the OR (95% CI) of the highest versus lowest quartile of EPA for AMD was 0.476 (0.304, 0.746, *P* for trend < 0.001). Meanwhile, DPA and DHA for Q4 versus Q1 were also negatively associated with any AMD. The inverse association was significant for DPA (OR: 0.467, 95% CI: 0.288–0.756, *P* for trend = 0.005) and DHA (OR: 0.586, 95% CI: 0.351–0.980, *P* for trend = 0.008).

**Table 3:** Multivariate logistic regression analysis of the association between dietary fatty acids and the odds of any AMD.

Variables	Continuous	Q1	Q2	Q3	Q4	*P* for trend
Energy density (mg/1000 kcal, mean ± SE)	OR (95% CI)		OR (95% CI)	OR (96% CI)	OR (97% CI)	
Total SFA	1.000 (1.000, 1.000)	Ref	0.984 (0.561, 1.728)	0.960 (0.571, 1.614)	1.070 (0.645, 1.774)	0.767
SFA 4:0 (Butanoic)	1.000 (0.999, 1.001)	Ref	1.050 (0.537, 2.053)	1.095 (0.618, 1.943)	1.076 (0.606, 1.913)	0.792
SFA 6:0 (Hexanoic)	1.001 (0.999, 1.002)	Ref	1.362 (0.689, 2.695)	1.075 (0.544, 2.126)	1.198 (0.669, 2.145)	0.791
SFA 8:0 (Octanoic)	0.999 (0.998, 1.001)	Ref	1.176 (0.656, 2.107)	1.285 (0.835, 1.979)	1.050 (0.629, 1.752)	0.983
SFA 10:0 (Decanoic)	1.000 (0.999, 1.002)	Ref	1.434 (0.806, 2.551)	0.938 (0.505, 1.741)	1.196 (0.689, 2.073)	0.866
SFA 12:0 (Dodecanoic)	1.000 (0.999, 1.000)	Ref	1.011 (0.568, 1.802)	1.182 (0.748, 1.869)	0.970 (0.577, 1.631)	0.804
SFA 14:0 (Tetradecanoic)	1.000 (1.000, 1.000)	Ref	1.110 (0.652, 1.893)	0.943 (0.588, 1.512)	1.232 (0.704,2.157)	0.455
SFA 16:0 (Hexadecanoic)	1.000 (1.000, 1.000)	Ref	0.887 (0.507, 1.554)	0.864 (0.483, 1.547)	1.225 (0.699, 2.148)	0.413
SFA 18:0 (Octadecanoic)	1.000 (1.000, 1.000)	Ref	0.971 (0.640, 1.472)	1.007 (0.697, 1.456)	1.194 (0.791, 1.803)	0.363
Total MUFA	1.000 (1.000, 1.000)	Ref	1.249 (0.730, 2.137)	1.098 (0.674, 1.788)	1.238 (0.776, 1.976)	0.427
MUFA 16:1 (Hexadecenoic)	1.000 (0.999, 1.001)	Ref	1.133 (0.738, 1.738)	1.150 (0.705, 1.878)	1.061 (0.660, 1.704)	0.814
MUFA 18:1 (Octadecenoic)	1.000 (1.000, 1.000)	Ref	1.146 (0.643, 2.042)	1.085 (0.678, 1.736)	1.194 (0.742, 1.921)	0.439
MUFA 20:1 (Eicosenoic)	1.001 (0.999, 1.002)	Ref	0.973 (0.621, 1.524)	1.034 (0.696, 1.536)	1.094 (0.725, 1.651)	0.538
MUFA 22:1 (Docosenoic)	1.000 (0.998, 1.002)	Ref	1.002 (0.661, 1.519)	0.801 (0.487, 1.316)	0.839 (0.540, 1.302)	0.436
Total PUFA	1.000 (1.000, 1.000)	Ref	1.341 (0.957, 1.880)	1.142 (0.758, 1.720)	0.933 (0.579, 1.502)	0.473
PUFA 18:2 (Octadecadienoic,n-6, LA)	1.000 (1.000, 1.000)	Ref	1.209 (0.848, 1.723)	1.137 (0.752, 1.719)	0.954 (0.583, 1.560)	0.687
PUFA 18:3 (Octadecatrienoic, n-3, ALA)	1.000 (0.999, 1.000)	Ref	0.882 (0.575, 1.355)	1.009 (0.572, 1.779)	0.839 (0.501, 1.404)	0.549
PUFA 18:4 (Octadecatetraenoic, n-3, SDA)	0.995 (0.985, 1.005)	Ref	0.934 (0.229, 3.819)	0.720 (0.476, 1.090)	0.772 (0.514, 1.161)	0.352
PUFA 20:4 (Eicosatetraenoic, n-6, AA)	0.997 (0.993, 1.000)	Ref	1.144 (0.774, 1.690)	0.863 (0.590, 1.262)	0.715 (0.418, 1.223)	0.117
PUFA 20:5 (Eicosapentaenoic, n-3, EPA)	0.996 (0.993, 0.999)^a^	Ref	0.919 (0.563, 1.500)	0.729 (0.458, 1.162)	0.476 (0.304, 0.746)^d^	< 0.001
PUFA 22:5 (Docosapentaenoic, n-3, DPA)	0.976 (0.962, 0.990)^b^	Ref	0.728 (0.447, 1.188)	0.718 (0.440, 1.174)	0.467 (0.288, 0.756)^e^	0.005
PUFA 22:6 (Docosahexaenoic, n-3, DHA)	0.996 (0.994, 0.999)^c^	Ref	1.339 (0.841, 2.134)	0.737 (0.467, 1.161)	0.586 (0.351, 0.980)^f^	0.008

Model was adjusted age, sex, ethnicity, BMI (continuous), smoking, alcohol, energy intake, marital status, education level, Hyperlipidemia, DM, CVD and hypertension.

^a^*P* value: 0.018; ^b^*P* value: 0.002; ^c^*P* value: 0.003; ^d^*P* value: 0.003; ^e^*P* value: 0.004; ^f^*P* value: 0.043.

### Association between dietary fat acids and AMD stages

We further explored the relationship between the FA intake and different AMD stages. For early AMD, after full adjustment, EPA (OR: 0.996, 95% CI: 0.992–0.999, *P* = 0.028), DPA (OR: 0.974, 95% CI: 0.961–0.988, *P* = 0.001) and DHA (OR: 0.996, 95% CI: 0.993–0.999, *P* = 0.006) were still significantly negatively associated with the odds of early AMD (Table 4). After categorizing FAs into quartiles, the negative association was significant for Q4 versus Q1 in EPA (OR: 0.461, 95% CI: 0.278–0.763, *P* for trend = 0.002), DPA (OR: 0.467, 95% CI: 0.275–0.795, *P* for trend = 0.006) and DHA (OR: 0.578, 95% CI: 0.342–0.975, *P* for trend = 0.007). Restricted cubic spline did not support a non-linear association between EPA, DPA, DHA consumption and odds of early AMD (all *P* non-linearity > 0.05, Supplementary Fig. 1). For late AMD, after full adjustment, 19 types of FAs were not significantly associated with the odds of AMD for either continuous or quartile data (Table 5). In sensitivity analysis, 4818 individuals were included after excluding 583 participants. The above-mentioned association between dietary FA intake and early AMD remains significant (Supplementary Table 1).

**Table 4:** Multivariate logistic regression analysis of the association between dietary fatty acids and early AMD.

Variables	Continuous	Q1	Q2	Q3	Q4	*P* for trend
Energy density (mg/1000 kcal, mean ± SE)	OR (95% CI)		OR (95% CI)	OR (96% CI)	OR (97% CI)	
Total SFA	1.000 (1.000, 1.000)	Ref	1.040 (0.584, 1.851)	1.065 (0.601, 1.886)	1.180 (0.670, 2.077)	0.535
SFA 4:0 (Butanoic)	1.000 (0.999, 1.001)	Ref	1.116 (0.561, 2.219)	1.099 (0.618, 1.953)	1.170 (0.637, 2.149)	0.619
SFA 6:0 (Hexanoic)	1.001 (0.999, 1.003)	Ref	1.417 (0.711, 2.821)	1.174 (0.582, 2.368)	1.360 (0.737, 2.508)	0.472
SFA 8:0 (Octanoic)	1.000 (0.998, 1.002)	Ref	1.256 (0.718, 2.195)	1.500 (0.935, 2.407)	1.219 (0.698, 2.131)	0.572
SFA 10:0 (Decanoic)	1.001 (0.999, 1.002)	Ref	1.479 (0.787, 2.780)	1.070 (0.555, 2.063)	1.379 (0.757, 2.512)	0.467
SFA 12:0 (Dodecanoic)	1.000 (0.999, 1.000)	Ref	1.004 (0.557, 1.813)	1.379 (0.853, 2.229)	1.107 (0.631, 1.944)	0.716
SFA 14:0 (Tetradecanoic)	1.000 (1.000, 1.000)	Ref	1.184 (0.637, 2.200)	0.959 (0.599, 1.535)	1.366 (0.735, 2.541)	0.311
SFA 16:0 (Hexadecanoic)	1.000 (1.000, 1.000)	Ref	0.856 (0.482, 1.520)	0.907 (0.503, 1.635)	1.288 (0.710, 2.336)	0.316
SFA 18:0 (Octadecanoic)	1.000 (1.000, 1.000)	Ref	1.036 (0.642, 1.672)	1.171 (0.789, 1.738)	1.365 (0.875, 2.127)	0.163
Total MUFA	1.000 (1.000, 1.000)	Ref	1.213 (0.737, 1.997)	1.159 (0.674, 1.994)	1.292 (0.826, 2.022)	0.285
MUFA 16:1 (Hexadecenoic)	1.000 (0.999, 1.001)	Ref	1.075 (0.680, 1.700)	1.198 (0.721, 1.990)	1.102 (0.672, 1.806)	0.627
MUFA 18:1 (Octadecenoic)	1.000 (1.000, 1.000)	Ref	1.122 (0.655, 1.924)	1.138 (0.669, 1.935)	1.251 (0.799, 1.959)	0.284
MUFA 20:1 (Eicosenoic)	1.001 (0.999, 1.003)	Ref	1.096 (0.711,1.688)	1.082 (0.717, 1.631)	1.142 (0.776, 1.681)	0.471
MUFA 22:1 (Docosenoic)	1.000 (0.998, 1.002)	Ref	0.965 (0.606,1.534)	0.815 (0.488, 1.363)	0.856 (0.535, 1.369)	0.558
Total PUFA	1.000 (1.000, 1.000)	Ref	1.453 (1.003,2.106)	1.137 (0.662, 1.953)	0.980 (0.575, 1.671)	0.565
PUFA 18:2 (Octadecadienoic, n-6, LA)	1.000 (1.000, 1.000)	Ref	1.368 (0.929,2.015)	1.068 (0.623, 1.830)	1.078 (0.628, 1.851)	0.898
PUFA 18:3 (Octadecatrienoic, n-3, ALA)	1.000 (0.999, 1.000)	Ref	0.970 (0.604,1.560)	1.108 (0.615, 1.995)	0.885 (0.509, 1.540)	0.666
PUFA 18:4 (Octadecatetraenoic, n-3, SDA)	0.993 (0.982, 1.004)	Ref	1.009 (0.242,4.214)	0.769 (0.505, 1.173)	0.739 (0.486, 1.123)	0.209
PUFA 20:4 (Eicosatetraenoic, n-6, AA)	0.996 (0.992,1.000)	Ref	1.180 (0.786,1.772)	0.809 (0.559, 1.171)	0.733 (0.428, 1.258)	0.12
PUFA 20:5 (Eicosapentaenoic, n-3, EPA)	0.996 (0.992,0.999)^a^	Ref	0.895 (0.546,1.468)	0.789 (0.493, 1.262)	0.461 (0.278, 0.763)^d^	0.002
PUFA 22:5 (Docosapentaenoic, n-3, DPA)	0.974 (0.961,0.988)^b^	Ref	0.776 (0.451,1.337)	0.774 (0.465, 1.290)	0.467 (0.275, 0.795)^e^	0.006
PUFA 22:6 (Docosahexaenoic, n-3, DHA)	0.996 (0.993,0.999)^c^	Ref	1.362 (0.883,2.100)	0.786 (0.497, 1.243)	0.578 (0.342, 0.975)^f^	0.007

Model was adjusted age, sex, ethnicity, BMI (continuous), smoking, alcohol, energy intake, marital status, education level, Hyperlipidemia, DM, CVD and hypertension.

^a^*P* value: 0.028; ^b^*P* value: 0.001; ^c^*P* value: 0.006; ^d^*P* value: 0.005; ^e^*P* value: 0.008; ^f^*P* value: 0.041.

**Table 5:** Multivariate logistic regression analysis of the association between dietary fatty acids and late AMD.

Variables	Continuous	Q1	Q2	Q3	Q4	*P* for trend
Energy density (mg/1000 kcal, mean ± SE)	OR (95% CI)		OR (95% CI)	OR (96% CI)	OR (97% CI)	
Total SFA	1.000 (1.000, 1.000)	Ref	0.797 (0.227, 2.798)	0.542 (0.196, 1.501)	0.642 (0.219, 1.884)	0.281
SFA 4:0 (Butanoic)	1.000 (0.998, 1.001)	Ref	0.788 (0.222, 2.798)	1.259 (0.324, 4.888)	0.725 (0.286, 1.837)	0.577
SFA 6:0 (Hexanoic)	0.998 (0.995, 1.001)	Ref	1.344 (0.353, 5.115)	0.665 (0.188, 2.345)	0.599 (0.249, 1.443)	0.105
SFA 8:0 (Octanoic)	0.998 (0.994, 1.001)	Ref	1.116 (0.334, 3.729)	0.452 (0.149, 1.370)	0.552 (0.208, 1.464)	0.089
SFA 10:0 (Decanoic)	0.999 (0.997, 1.001)	Ref	1.661 (0.524, 5.263)	0.379 (0.142, 1.013)	0.583 (0.216, 1.574)	0.054
SFA 12:0 (Dodecanoic)	1.000 (0.999, 1.000)	Ref	1.130 (0.351, 3.636)	0.429 (0.168, 1.096)	0.467 (0.145, 1.504)	0.112
SFA 14:0 (Tetradecanoic)	1.000 (0.999, 1.001)	Ref	0.934 (0.335, 2.605)	1.124 (0.336, 3.762)	0.685 (0.276, 1.705)	0.424
SFA 16:0 (Hexadecanoic)	1.000 (1.000, 1.000)	Ref	1.076 (0.375, 3.089)	0.671 (0.197, 2.291)	0.840 (0.289, 2.440)	0.58
SFA 18:0 (Octadecanoic)	1.000 (0.999, 1.000)	Ref	0.720 (0.249, 2.081)	0.345 (0.099, 1.203)	0.522 (0.184, 1.481)	0.121
Total MUFA	1.000 (1.000, 1.000)	Ref	1.788 (0.581, 5.506)	0.748 (0.287, 1.950)	0.924 (0.231, 3.691)	0.542
MUFA 16:1 (Hexadecenoic)	0.999 (0.998, 1.001)	Ref	1.748 (0.629, 4.853)	0.893 (0.331, 2.410)	0.689 (0.163, 2.913)	0.409
MUFA 18:1 (Octadecenoic)	1.000 (1.000, 1.000)	Ref	1.519 (0.485, 4.760)	0.789 (0.321, 1.941)	0.851 (0.210, 3.454)	0.541
MUFA 20:1 (Eicosenoic)	1.000 (0.995, 1.005)	Ref	0.341 (0.107, 1.084)	0.838 (0.300, 2.339)	0.951 (0.292, 3.102)	0.89
MUFA 22:1 (Docosenoic)	0.999 (0.994, 1.005)	Ref	1.547 (0.566, 4.227)	0.858 (0.221, 3.324)	0.759 (0.253, 2.281)	0.34
Total PUFA	1.000 (1.000, 1.000)	Ref	0.535 (0.180, 1.591)	1.511 (0.473, 4.830)	0.700 (0.213, 2.300)	0.916
PUFA 18:2 (Octadecadienoic,n-6, LA)	1.000 (1.000, 1.000)	Ref	0.331 (0.112, 0.980)	1.924 (0.571, 6.485)	0.285 (0.118, 0.688)^a^	0.297
PUFA 18:3 (Octadecatrienoic, n-3, ALA)	0.999 (0.998, 1.000)	Ref	0.454 (0.150, 1.374)	0.545 (0.214, 1.388)	0.602 (0.236, 1.538)	0.411
PUFA 18:4 (Octadecatetraenoic, n-3, SDA)	1.012 (0.994, 1.030)	Ref	0.467 (0.136, 0.891)	0.514 (0.204, 1.291)	1.353 (0.500, 3.663)	0.28
PUFA 20:4 (Eicosatetraenoic, n-6, AA)	1.000 (0.993, 1.006)	Ref	0.687 (0.234, 2.012)	1.429 (0.550, 3.711)	0.608 (0.205, 1.803)	0.649
PUFA 20:5 (Eicosapentaenoic, n-3, EPA)	0.999 (0.990, 1.007)	Ref	1.117 (0.405, 3.080)	0.268 (0.076, 0.945)	0.682 (0.204, 2.284)	0.626
PUFA 22:5 (Docosapentaenoic, n-3, DPA)	0.988 (0.939, 1.040)	Ref	0.424 (0.153, 1.174)	0.383 (0.127, 1.153)	0.564 (0.164, 1.939)	0.484
PUFA 22:6 (Docosahexaenoic, n-3, DHA)	0.999 (0.994, 1.005)	Ref	0.949 (0.328, 2.743)	0.396 (0.141, 1.114)	0.718 (0.255, 2.021)	0.551

Model was adjusted age, sex, ethnicity, BMI (continuous), smoking, alcohol, energy intake, marital status, education level, Hyperlipidemia, DM, CVD and hypertension.

^a^*P* value: 0.009.

## Discussion

We presented a nationwide cross-sectional study from the US NHANES (2005–2008) assessing the associations between dietary SFA, MUFA and PUFA intake and the odds of any AMD. This study suggested that higher dietary n-3 PUFAs in the form of EPA, DPA and DHA are associated with a decreased odds of any AMD. Meanwhile, the total SFAs, MUFAs, PUFAs and 16 other individual FAs were not associated with the odds of any AMD. Subgroup analysis showed that increased intake of EPA, DPA and DHA was inversely associated with the odds of early AMD. However, no clear association was found between specific types of FAs and late AMD.

This investigation builds on previous studies that have explored the associations between the intake of different types of dietary FAs and AMD risk^9,12,19,20,28,29^. Most of these studies showed that n-3 PUFAs were significantly associated with a reduced risk of AMD^12,30,31^. Christen et al. noted an approximately 38% reduction in early AMD risk for DHA and a 34% reduction for EPA in the Women's Health Study^12^. A recent meta-analysis suggested that dietary higher DHA and EPA intake were significantly associated with 50% and 60% reductions in early AMD risk, respectively^32^. Although the supplement of n-3 PUFAs to the AREDS formulation was not further decrease the risk for AMD, it is believed that higher doses of EPA and DHA may have a beneficial effect^9^. The study supported previous studies showing that higher DHA and EPA intake inversely associated with the odds of AMD. There are some potential explanations for this discrepancy. van Leeuwen et al. noted that the control group in AREDS2 was not representative of the general population, as more than 11.1% of subjects in the control groups took n-3 PUFAs on their own, leading the main result towards the null^33^. Additionally, it is also possible that FAs other than DHA and EPA, such as DPA, which is often consumed with DHA and EPA but is not rich in the AREDS2 DHA/EPA formulation, may be responsible for null finding^34^.

Our results also suggested that higher DPA intake was negatively associated with the odds of AMD. Few studies have reported the relationship between dietary DPA and AMD risk. But some studies have reported the plasma levels of DPA may relate to AMD risk. Higher macular pigment optical density in subjects in the Limpia study was found to be significantly correlated with higher plasma levels of DPA rather than DHA or EPA^35^. A recent study conducted in China discovered the levels of circulating DPA was lower in neovascular AMD as well^36^. Most n-3 PUFA studies have attributed the beneficial effects to DHA and EPA. Since DPA is taken as a biological reservoir of DHA and EPA^37^. In recent years, there has been an increasing amount of research supporting the fact that DPA is a bioactive fatty acid that provides health benefits to humans^38–40^. In vitro cell experiments showed that DPA could down-regulate the mRNA expression of pro-inflammatory factors^41^. The main source of the decrease in inflammation related to DPA appears to be these lipid metabolites derived from DPA^42^. The suggested mechanisms involve DPA-derived lipid metabolites with anti-inflammatory properties that may benefit people with AMD, since inflammation appears to have a pivotal role in pathological processes in AMD^43^. It would be interesting to further investigate the impact of dietary DPA on AMD.

With regard to MUFAs or SFAs, their associations with AMD were not consistent across epidemiological studies, and few showed significant results^21,44–46^. Early US cohorts have demonstrated a positive association between MUFA or SFA intake and AMD risk^16,46^ and a few studies have reported a negative association between MUFA or SFA intake and AMD risk in Portuguese and Japanese populations^19,20^. In this study, neither total SFA, MUFA, nor individual SFA, MUFA intake was associated with the odds of AMD in the US population. These inconsistencies may reflect that different races have different dietary patterns. Compared with Western populations, which consume more meat and dairy foods, the Asian diet consumes less SFA-containing foods, and the Mediterranean diet consumes more plant food and olive-related products which contain rich MUFAs^47,48^. To date, the relationship between MUFAs and SFAs intake and AMD risk is still unclear, which may be due to their multidimensional nature and complicated metabolic pathways^29,33^.

Although numerous studies have shown a protective effect of dietary n-3 PUFAs with early AMD, the results for late AMD were inconsistent^21,34,49,50^. This study showed similar results to a recent meta-analysis that demonstrated that higher intake of n-3 PUFAs did not reduce the risk of late AMD^32^. A prospective control study in the US, including 75,889 women and 38,961 men, indicated that higher intakes of EPA and DHA were not associated with late AMD^11^. Meanwhile, a previous randomized intervention trial (Nutritional AMD Treatment-2) suggested that oral supplementation with DHA and EPA indicated no significant difference in neovascular incidence^18^. Mitchell et al. pointed out that the relatively low incidence of only 5% of early AMD cases may develop into late AMD in 5 years of observation, which may contribute to the null findings^1^. Additionally, studies suggest that some pathophysiological pathways of GA and neovascular AMD are partially distinct^34^. This study failed to separate late AMD into GA and vascular AMD, which could have confused the results. Therefore, further studies are required to provide stronger evidence for recommendations.

The current study possesses various strengths, such as a large sample that represents the entire population of the United States and a comprehensive assessment of a wide range of dietary FAs. However, there are several limitations to this current study. First, the cross-sectional study design cannot further explore the causal association. Second, the NHANES database did not provide information on late AMD subtype. It was not possible to determine the association between FAs and the subtype of late AMD. Third, since fundus data were not collected in other years of NHANES, dietary intake data were still obtained from NHANES 2005–2008 and could not represent dietary changes in later years. Finally, despite a rigorous adjustment for potential confounders, the potential for residual confounding cannot be excluded. When interpreting these findings, it is important to consider that residual confounding may introduce some bias into the results.

In summary, this study examined the association between the intake of 19 dietary FAs and AMD in the 2005–2008 NHANES. The results suggested that higher dietary intake of DHA, DPA, and EPA is inversely associated with the odds of any AMD, particularly early AMD, in the US population. The study supported previous research indicating that n-3 PUFAs are benefit patients with AMD. However, the lack of association between other FAs and AMD and the inconsistent relationship between n-3 PUFAs and late AMD require further investigation. Therefore, future well-designed prospective studies are warranted to verify these findings and provide additional evidence for dietary recommendations and interventions for AMD prevention and management.

### Supplementary Information

Supplementary Information.

Supplementary Table 1.

Supplementary Figure 1.

## Data Availability

All data used in this study are available in NHANES website: https://www.cdc.gov/nchs/nhanes/ and in supplementary file.
