# Supplementary material for: Association between dietary consumption of fatty acids and age-related macular degeneration in the National Health and Nutrition Examination Survey
Source: Sci Rep. 2024 May 14;14:11016. doi: 10.1038/s41598-024-61833-6 (PMC11094158; doi:10.1038/s41598-024-61833-6)
Supplement: Supplementary file 2 — Supplementary Table 1. [file 41598_2024_61833_MOESM2_ESM.docx]

Supplementary table 1: Multivariate logistic regression analysis of the association between dietary fatty acids and early AMD.

| Variables | Continuous | Q1 | Q2 | Q3 | Q4 | *P* for trend |
| --- | --- | --- | --- | --- | --- | --- |
| Energy density (mg/1,000 kcal, mean ± SE) | OR (95% CI) |  | OR (95% CI) | OR (96% CI) | OR (97% CI) |  |
| Total SFA | 1.000 (1.000,1.000) | ref | 1.000 (0.536,1.867) | 1.023 (0.570,1.837) | 1.125 (0.637,1.987) | 0.626 |
| SFA 4:0 (Butanoic) | 1.080 (1.061,1.099) | ref | 1.073 (0.531,2.168) | 1.053 (0.603,1.841) | 1.125 (0.623,2.032) | 0.680 |
| SFA 6:0 (Hexanoic) | 1.001 (0.999,1.003) | ref | 1.346 (0.661,2.740) | 1.137 (0.560,2.308) | 1.297 (0.712,2.360) | 0.523 |
| SFA 8:0 (Octanoic) | 1.000 (0.998,1.002) | ref | 1.199 (0.681,2.110) | 1.446 (0.877,2.383) | 1.162 (0.673,2.008) | 0.653 |
| SFA 10:0 (Decanoic) | 1.000 (0.999,1.002) | ref | 1.414 (0.714,2.802) | 1.030 (0.525,2.018) | 1.321 (0.723,2.411) | 0.517 |
| SFA 12:0 (Dodecanoic) | 1.000 (0.999,1.000) | ref | 0.965 (0.527,1.765) | 1.336 (0.821,2.175) | 1.062 (0.597,1.889) | 0.805 |
| SFA 14:0 (Tetradecanoic) | 1.000 (1.000,1.000) | ref | 1.139 (0.596,2.180) | 0.931 (0.578,1.497) | 1.306 (0.707,2.414) | 0.359 |
| SFA 16:0 (Hexadecanoic) | 1.000 (1.000, 1.000) | ref | 0.833 (0.462,1.500) | 0.876 (0.489,1.569) | 1.239 (0.694,2.212) | 0.358 |
| SFA 18:0 (Octadecanoic) | 1.000 (1.000, 1.000) | ref | 1.063 (0.662,1.708) | 1.181 (0.784,1.781) | 1.359 (0.858,2.153) | 0.172 |
| Total MUFA | 1.000 (1.000, 1.000) | ref | 1.205 (0.717,2.026) | 1.193 (0.689,2.064) | 1.267 (0.788,2.035) | 0.317 |
| MUFA 16:1 (Hexadecenoic) | 1.000 (0.999,1.001) | ref | 1.033 (0.625,1.706) | 1.163 (0.693,1.953) | 1.057 (0.630,1.776) | 0.731 |
| MUFA 18:1 (Octadecenoic) | 1.000 (1.000, 1.000) | ref | 1.114 (0.635,1.953) | 1.161 (0.679,1.985) | 1.239 (0.776,1.979) | 0.296 |
| MUFA 20:1 (Eicosenoic) | 1.001 (0.999,1.003) | ref | 1.106 (0.704,1.737) | 1.080 (0.710,1.641) | 1.189 (0.802,1.764) | 0.341 |
| MUFA 22:1 (Docosenoic) | 1.000 (0.998,1.002) | ref | 0.951 (0.584,1.547) | 0.814 (0.478,1.386) | 0.877 (0.539,1.426) | 0.677 |
| Total PUFA | 1.000 (1.000, 1.000) | ref | 1.438 (0.989,2.092) | 1.131 (0.643,1.988) | 1.003 (0.578,1.741) | 0.638 |
| PUFA 18:2 (Octadecadienoic,n-6, LA) | 1.000 (1.000, 1.000) | ref | 1.346 (0.898,2.016) | 1.058 (0.605,1.850) | 1.097 (0.629,1.912) | 0.968 |
| PUFA 18:3 (Octadecatrienoic, n-3, ALA) | 1.000 (0.999,1.000) | ref | 0.964 (0.592,1.567) | 1.104 (0.603,2.020) | 0.902 (0.513,1.584) | 0.728 |
| PUFA 18:4 (Octadecatetraenoic, n-3, SDA) | 0.994 (0.983,1.005) | ref | 0.957 (0.198,4.619) | 0.774 (0.501,1.197) | 0.763 (0.489,1.192) | 0.299 |
| PUFA 20:4 (Eicosatetraenoic, n-6, AA) | 0.996 (0.992,1.000) | ref | 1.205 (0.811,1.789) | 0.821 (0.560,1.203) | 0.708 (0.402,1.246) | 0.095 |
| PUFA 20:5 (Eicosapentaenoic, n-3, EPA) | 0.997 (0.993,1.000)^a^ | ref | 0.899 (0.541,1.494) | 0.780 (0.474,1.282) | 0.491 (0.294,0.823)^d^ | 0.007 |
| PUFA 22:5 (Docosapentaenoic, n-3, DPA) | 0.977 (0.962,0.992)^b^ | ref | 0.807 (0.468,1.391) | 0.787 (0.468,1.322) | 0.510 (0.296,0.879)^e^ | 0.014 |
| PUFA 22:6 (Docosahexaenoic, n-3, DHA) | 0.996 (0.993,1.000)^c^ | ref | 1.364 (0.871,2.135) | 0.789 (0.492,1.265) | 0.603 (0.348,1.045)^f^ | 0.017 |

Model was adjusted age, sex, ethnicity, BMI (continuous), smoking, alcohol, energy intake, marital status, education level, Hyperlipidemia, DM, CVD and hypertension.

a: P value: 0.08; b: P value: 0.005; c: P value: 0.032, d: P value: 0.012, e: P value: 0.02, f: P value: 0.067.
