# Supplementary material for: Association between dietary consumption of fatty acids and age-related macular degeneration in the National Health and Nutrition Examination Survey
Source: Sci Rep. 2024 May 14;14:11016. doi: 10.1038/s41598-024-61833-6 (PMC11094158; doi:10.1038/s41598-024-61833-6)

Supplementary Figure 1: Dose-response relationship between EPA, DPA, DHA consumption and early AMD in NHANES. Covariates were fully adjusting for age, sex, ethnicity, BMI (continuous), smoking, alcohol, energy intake, marital status, education and medical history. Solid red lines indicate estimates, and shadow depict 95% CI.


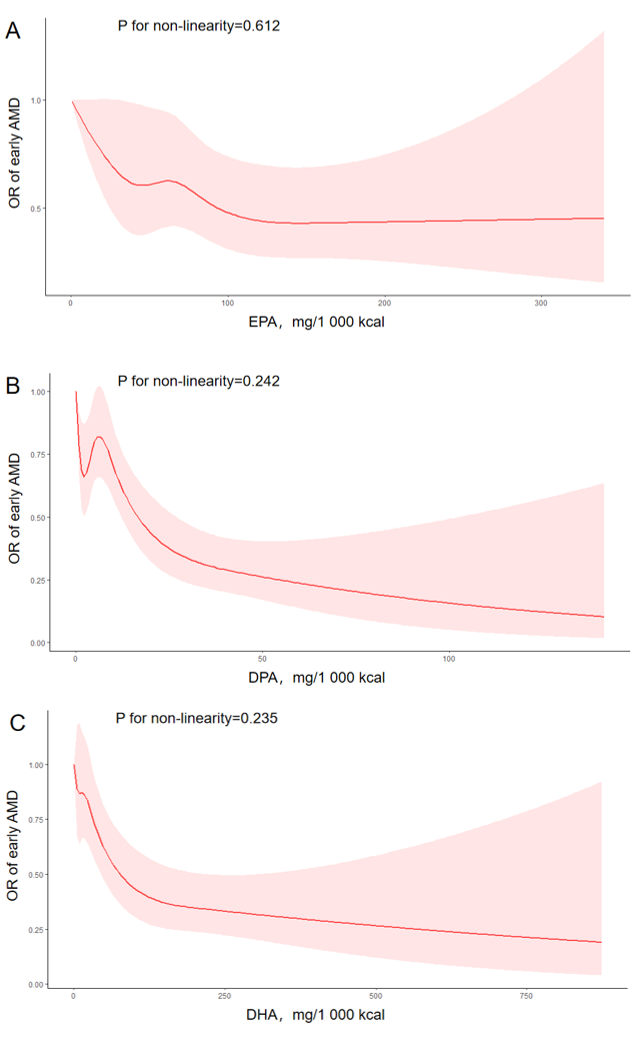

Supplement: Supplementary file 3 — Supplementary Figure 1. [file 41598_2024_61833_MOESM3_ESM.docx]
